# 422. Sustainability of Improvements to Hand Hygiene Performance Throughout the COVID-19 Pandemic

**DOI:** 10.1093/ofid/ofab466.622

**Published:** 2021-12-04

**Authors:** Victoria R Williams, Matthew Muller, Jeff Powis, Daniel R Ricciuto, Dominik Mertz, Kevin Katz, Lucas Castellani, Susy S Hota, Susy S Hota, Michael Payne, Jennie Johnstone, Jerome A Leis

**Affiliations:** 1 Sunnybrook Health Sciences Centre, Toronto, ON, Canada; 2 St. Michael’s Hospital, Toronto, Ontario, Canada; 3 University of Toronto, Toronto, ON, Canada; 4 Lakeridge Health, Oshawa, Ontario, Canada; 5 Hamilton Health Sciences, McMaster University, Hamilton, ON, Canada; 6 North York General Hospital, Toronto, ON, Canada; 7 Sault Area Hospital, Sault Ste Marie, Ontario, Canada; 8 University Health Network, Toronto, ON, Canada; 9 London Health Sciences Centre, London, Ontario, Canada; 10 Sinai Health System, Toronto, Ontario, Canada

## Abstract

**Background:**

Hand hygiene (HH) is a standard infection prevention and control precaution to be applied in healthcare settings to prevent transmission of COVID-19. Many healthcare institutions observed significant improvements in HH performance during wave one of the COVID-19 pandemic but the sustainability of this change is unknown. Our aim was to evaluate long-term HH performance throughout subsequent waves of the pandemic across acute care hospitals in Ontario, Canada.

**Methods:**

HH adherence was measured using a previously validated group electronic monitoring system which was installed on all alcohol handrub and sink soap dispensers inside and outside each patient room across 56 inpatient units (35 wards and 21 critical care units) spanning 13 acute care hospitals (6 university and 7 community teaching hospitals) from 1 November 2019 to 31 May 2021. Daily HH adherence was compared with daily COVID-19 case count across Ontario. During this period, weekly performance continued to be reported to units but unit-based quality improvement discussions were inconsistent due to the COVID-19 response.

**Results:**

Figure 1 depicts daily aggregate HH adherence plotted against the new daily COVID-19 case count across Ontario. An elevation in HH adherence was seen prior to the start of the first wave, rising almost to 80% and then remained above 70% for the peak of wave one. During waves two and three, peak COVID-19 case counts were associated with a maximum HH adherence of 51%, only marginally above the pre-pandemic baseline. After the end of wave one (from 1 July 2020 to 31 May 2021) the median HH performance was only 49% (interquartile range 47%-50%).

Figure 1. Hand hygiene adherence across 13 acute care hospitals in comparison to overall new daily COVID-19 cases in Ontario

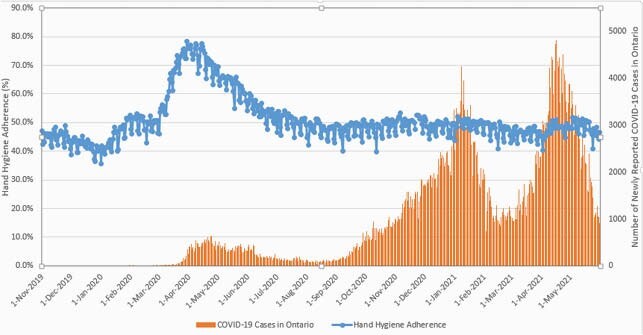

**Conclusion:**

Initial improvements in HH adherence preceding the start of the COVID-19 pandemic were not sustained, possibly due to increasing comfort and reduced anxiety associated with providing care to COVID-19 patients leading to a perception of reduced COVID-19 transmission risk. These findings highlight the need for HH monitoring to be tied to longitudinal unit-led quality improvement in order to achieve durable changes in practice.

**Disclosures:**

**Susy S. Hota, MSc MD FRCPC**, **Finch Therapeutics** (Research Grant or Support) **Susy S. Hota, MSc MD FRCPC**, Finch Therapeutics (Individual(s) Involved: Self): Grant/Research Support

